# Real-time monitoring of glycocarrier formation unravels cryptic details in glycosyl transfer

**DOI:** 10.1039/d6cb00069j

**Published:** 2026-06-01

**Authors:** Ryan D. Packer, Angelo Gallo, Alexander D. Cameron, Somnath Mondal, Józef R. Lewandowski, Manuela Tosin

**Affiliations:** a Department of Chemistry, University of Warwick, Gibbet Hill Coventry CV4 7AL UK m.tosin@warwick.ac.uk; b School of Life Sciences, Gibbet Hill Campus Coventry CV4 7AL UK; c Department of Chemistry, University of Turin, Via Pietro Giuria 42 Torino (TO) Italy

## Abstract

Glycosyl transfer constitutes a key step in the functionalisation of bioactive molecules, including proteins and natural products. *In vitro* studies to dissect glycosyltransferase catalysis are often challenging due to poor enzyme solubility, enzyme instability, and the requirement of advanced analytical setups. Herein, we report the development and use of a polynucleotide phosphorylase (PNPase)-coupled assay for the real-time monitoring of mannosyl transfer to polyprenyl phosphate carriers. This, together with molecular modeling, dynamics and site-directed mutagenesis, has allowed us to gather in-depth insights into enzyme catalysis associated with health and disease.

## Abstract 2

A real-time fluorescence-based assay has been developed to unveil molecular details of glycocarrier formation in health and disease.

## Graphical abstract

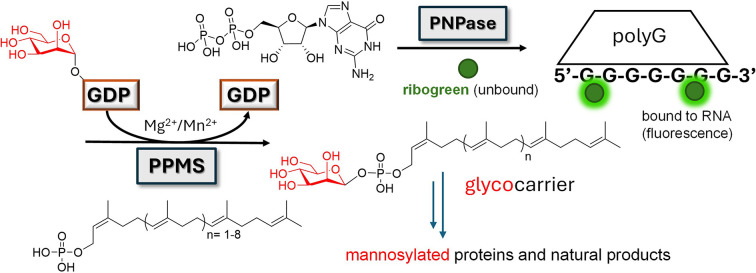

## Introduction

**Fig. 1:**
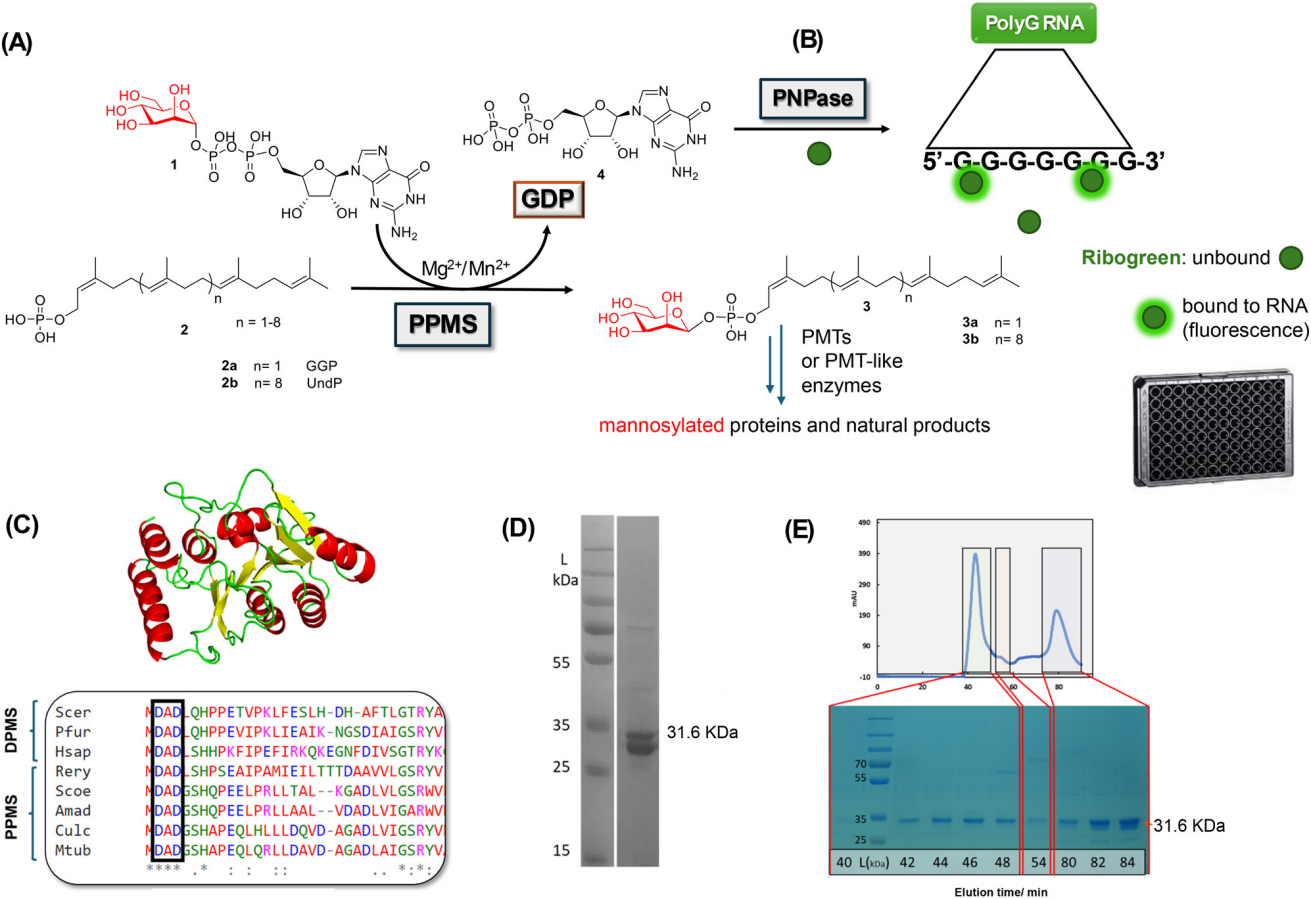
(A) Formation of phosphate polyprenyl glycocarriers (3) catalysed by a polyprenyl phosphomannose synthase (PPMS).^3^ (B) Newly devised detection of PPMS activity (this work): a PNPase enzyme converts the released GDP byproduct (4) to polyG RNA, which is complexed with Ribogreen to generate fluorescence^15^ (read in 96-well black plates; details are given in the Materials and methods section and in the SI). (C) Homology model of a newly identified *C. ulcerans* PPMS (this work), and partial sequence alignment of selected PPMSs (Rery, Scoe, Amad, Culc and Mtb) and DPMSs (Scer, Pfur, and Hsap), highlighting the conserved D × D motif within GT-2 enzymes (Culc is *C. ulcerans* PPMS; for further details about this and other PPMS/DPMS sequences, see Fig. S1). (D) Recombinant hexahistidine-tagged *C. ulcerans* sp. PPMS (CU PPMS) expressed and IMAC-purified (right lane) from *E. coli*, and (E) size-exclusion chromatogram of the latter, with accompanying 10% SDS-PAGE analysis: the upper band (31.6 kDa) alone elutes as an aggregate, whereas two bands (of 31.6 and 27.8 kDa, respectively, SI) co-elute as a dimeric mixture, which is catalytically active (SI).

Carbohydrate moieties are ubiquitous in nature and play several roles associated with energy provision, structural support and information exchange. Glycosyltransferase enzymes (GTs)^1^ are responsible for the functionalisation of macromolecules, such as proteins, and small molecules, such as natural products, with the presence of carbohydrates determining biomolecule localisation, conformation and activity. In biological glycosyl transfer, donor molecules include mostly nucleotide diphosphate (*e.g.* for Leloir glycosyltransferases)^2^ and lipid phosphate sugars.^3^ Acceptor molecules comprise nucleophilic residues, such as hydroxyl, amino, thiol, and phosphate groups; glycosylation of carbon atoms such as the C2 of tryptophan is also known.^4^ Currently, there are 140 GT families in the Carbohydrate-Active enZYmes (CAZY) database, most of which remain under investigation and are underexploited. Amongst them, polyprenyl phosphomannose synthases (PPMSs) utilise GDP-mannose (1) and polyprenyl phosphates (2) to form β-mannosylated polyprenyl phosphates (3, Fig. 1A); these act as glycocarriers and donors for protein mannosylation at the membrane–water interface, as well as for the late stage functionalisation of natural products in Gram positive bacteria and mycobacteria.^5^ PPMSs belong to the GT2 family: they feature a GT-A Rossmann-like fold and a DXD motif for divalent cation and sugar nucleotide binding (Fig. 1C).^6^ Their structures greatly vary with organism complexity (Fig. S1) and feature a cytoplasmatic catalytic domain linked to a highly hydrophobic membrane-associated or membrane-bound component. In eukaryotes and archaea, dolichyl phosphate (DolP) is present in place of polyprenyl phosphates. Enzymes catalysing the transfer of mannose from GDP to DolP are known as dolichyl phosphomannose synthases (DPMSs): similarly to (3), DPMS products deliver mannose to all glycosylation processes at the membrane–water interface, including N-, O-, C- and GPI anchor mannosylation.^7^ Because of their critical role in countless health and disease processes, most studies to date have focused on DPMS function *in vivo* involving gene editing and knock-out. Only in the past 20 years, bacterial PPMSs have become more widely known and have gathered interest for biotechnology and biomedical purposes. Indeed, PPMSs catalyse the direct formation of β-mannosyl linkages, which are synthetically challenging; moreover, PPMS malfunctioning or absence in bacteria has been implicated in increased susceptibility to antibiotics,^8^ and decreased cell viability and virulence,^9^ making PPMSs potential target candidates for novel antibacterial drugs. Reports on the crystal structures of an archaeal DPMS^10^ and of a bacterial polyprenyl phosphoglucose synthase^11^ have contributed to our understanding of PPMS workings. However, many details on their catalysis remain undetermined due to the poor solubility of recombinant enzymes, the need for complex and expensive substrates and the lack of straightforward methods to monitor their activity. To date, most studies on PPMSs (and DPMSs) have involved the use of radioactive substrates and TLC analyses, as well as coupled assays involving colorimetric detection of GDP (4), the byproduct of PPMS-catalysed reactions.^12^ However, all these suffer from some pitfalls. Detergent is required to solubilise most lipid phosphate acceptors, creating significant interference in the acquisition of activity data, as well as in direct product characterisation and detection by NMR, MS, HPLC, *etc.* In general, most GT assay methods^13^ provide single timepoint measurements, requiring some form of processing or ‘quenching’ to generate a signal and disrupting the enzyme activity itself. In some cases, high substrate concentrations are required to gather an output (*e.g.* colorimetric), and this can lead to enzyme inhibition or produce false positives. The cost of sensitive and reliable assay kits, reagents and output detection systems can be prohibitive for some applications, such as high-throughput screening, and outputs can be significantly affected by the presence of co-solvents such as DMSO and other small molecules commonly used in enzymatic assays.

In an attempt to develop a straightforward, low-cost, versatile and sensitive method to dissect PPMS catalysis *in vitro*, we envisaged that a polynucleotide phosphorylase (PNPase) enzyme could be used for the quantitative detection of GDP (4) generated by active PPMSs (Fig. 1B). In addition to acting as an exoribonuclease, PNPase can catalyse the formation of homopolymer RNA from all four commonly encountered nucleotide diphosphates (NDPs):^14^ the resulting polyRNA can be detected quantitatively and sensitively upon binding of a fluorescent dye.^15^ The use of PNPases in coupled assays has been reported for the study of phosphate releasing enzymes, based on the exoribonuclease activity of PNPases and changes in UV absorption of substrates involved in such activity.^15,16^ In one instance, *E. coli* PNPase has been used as a polymerase for the detection of ADP released by the ligase MurC, in conjunction with Ribogreen (commonly used for RNA detection).^17^ To the best of our knowledge, PNPases have yet to be used further as coupled polymerases or for the study of GTs.

## Results and discussion

A PNPase enzyme from *E. coli* was selected based on its high reported RNA formating activity from GDP.^15^ The *pnp* gene was cloned from *E. coli* TOP10 into a pET28a(+) vector encoding for an N-terminal hexahistidine tag, as reported in the supplementary information (SI). *E. coli* BL21 (DE3) cells transformed with the pET28a(+)-*pnp* plasmid were then used for PNPase overexpression upon induction with IPTG. The recombinant enzyme (of approximately 78 kDa) was isolated and purified in excellent yield (approx. 64 mg L^−1^) following Co^2+^ affinity chromatography (Fig. S2). Detection of PNPase activity was first assessed under conditions later used for glycosyltransferase activity (50 mM Tris-HCl, 50 mM NaCl, 1 mM MgCl_2_, 0.005% Triton X-100, 0.1 mM DTT, pH 7.5). Keeping the GDP amount constant and exciting at 488 nm, the fluorescence output at 512 nm derived from newly formed poly(G) RNA in complex with Ribogreen was measured as a function of increasing amounts of PNPase (SI and Fig. S2). From this, an optimal PNPase working concentration range (low µM) was established and a standard curve for GDP detection was built (Fig. S2). Mutants of the PNPase were generated by site-directed mutagenesis^18^ and tested under the same conditions to assert the authenticity of the fluorescent output deriving from the coupled polymerase activity (Fig. S3). Mutant N345D, featuring enhanced polymerisation activity,^18^ was subsequently utilised as the coupling enzyme to detect GT activity.

The PNPase–Ribogreen GDP detection method was then applied to investigate the workings of a newly identified soluble PPMS from *Corynebacterium ulcerans* (CU), an emerging pathogen responsible for diphtheria and diphtheria-like infections.^19^ This enzyme was initially identified through a search for soluble PPMS candidates and originally reported as the product of gene SQG58706 from a clinical sample of *P. aeruginosa* (a Gram-negative bacterial strain). However, upon further verification using BLAST and other bioinformatics tools, it was identified by us as belonging to *C. ulcerans*.^20^ A synthetic gene encoding for the *C. ulcerans* PPMS bearing a hexahistidine N-terminal tag was designed and purchased (SI). Protein expression in *E. coli* BL21 (DE3) cells transformed with the pET28a-*cuppms* plasmid was carried out; purification by immobilised Ni^2+^ affinity chromatography led to the isolation of the desired protein (31.6 kDa) together with a C-terminal truncation lacking the final 35 residues (27.4 kDa, Fig. 1D and Fig. S3A). Protein size-exclusion chromatography (SEC) of this mixture did not lead to its separation, with the mixture eluting in a dimeric form (Fig. 1E). This was also subjected to hydrophobic ionic chromatography (HIC), after which the lower size component (27.4 kDa) was partially isolated (Fig. S3C); however, this alone did not prove catalytically active. A CU PPMS Δ35 mutant was independently generated by site-directed mutagenesis and proved inactive (Fig. S3D). PPMSs and related enzymes have been found by us and others to mostly function as dimers.^21^

**Fig. 2:**
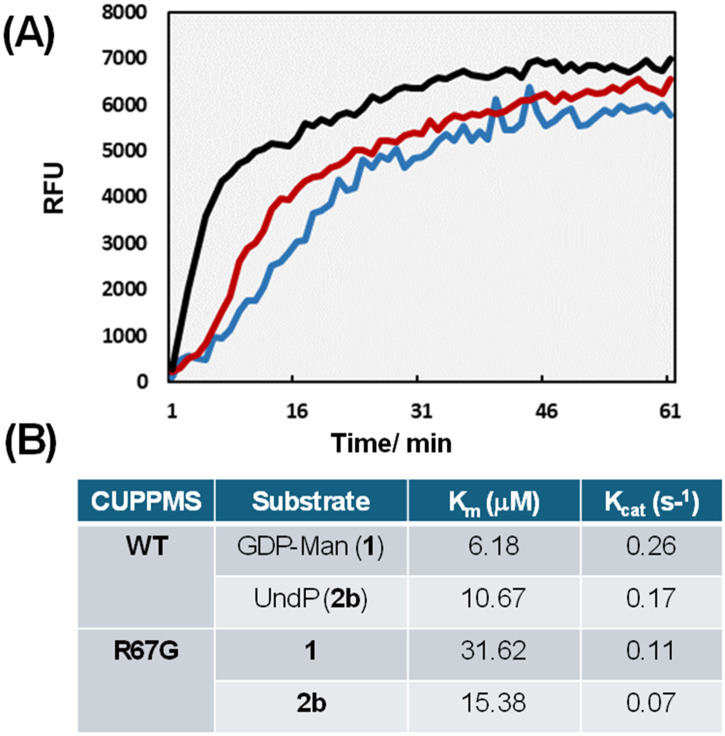
(A) Real-time fluorescence monitoring of *C. ulcerans* PPMS wild-type (WT, black), R67G (red) and R67A (blue) mutants incubated with GDP-mannose (1, 50 µM) and undecaprenyl phosphate (UndP, 2b, 50 µM) in 50 mM Tris·HCl, pH 7.5, 50 mM NaCl, 0.005% Triton X-100, 0.1 mM DTT, and 1 mM MgCl_2_ (further details are given in the SI); (B) kinetic parameters obtained for wild-type CUPPMS and mutant R67G (mimic of a DPMS mutant associated with disease)^24^ acquired *via* the PNPase/Ribogreen assay (further details are given in the Materials and methods section and the SI).

The CU PPMS was next investigated for functional studies in its dimeric mixture form. Upon incubation with GDP-mannose (1, 50 µM) and commercially available undecaprenyl phosphate (UndP, 2b, 50 µM) – commonly utilised in bacterial protein glycosylation – CU PPMS activity was monitored in real time using PNPase and Ribogreen. Under the tested conditions, the GT reaction reached equilibrium within 30–45 min (Fig. 2A, black trace), and a calculated substrate conversion of 60% based on released GDP. When a 4-fold excess of UndP was utilised, up to 85% conversion of GDP to β-mannosylated UndP (3b) was estimated (SI and Fig. S3E). Mannosyl phosphoisoprenoid formation was independently verified by scaled up assays utilising GDP-mannose (1) and synthetic phytanyl phosphate (PhytP, a saturated analogue of geranylgeranyl phosphate 2a, SI) for product extraction and characterisation (Fig. S3F).^22^ Kinetic parameters for GDP-mannose (1) and UndP (2b) were measured for the CU PPMS (Fig. 2B): *K*_m_ values were found to be in the micromolar range as for other PPMS and DPMS enzymes measured through other methods.^21,23,24^

Encouraged by our newly devised ability to monitor the *C. ulcerans* PPMS activity in real time, we further utilised our coupled assays to unravel the cryptic role of conserved protein residues in PPMS and DPMS catalysis. The mutation of R92 to a glycine in the human DPM1 synthase has been reported as inherently associated with congenital disorder of glycosylation CDG1e, a debilitating health condition characterised by brain and muscle development abnormalities.^24^ An arginine (R) residue at the same position (67 in our CU PPMS, untagged sequence) is conserved across all PPMSs and DPMSs (Fig. S4A). We therefore generated *C. ulcerans* PPMS R67G and R67A mutants by site-directed mutagenesis (SI) to investigate their activity. The results of these studies are illustrated in Fig. 2A and B. Compared to the wild-type (WT) *C. ulcerans* enzyme, both R67G and R67A mutants (featuring a similar size-exclusion chromatography profile to the WT enzyme, Fig. S4) displayed much reduced activity and higher *K*_m_ values against GDP-mannose. These values very closely mirror the *K*_m_ values calculated for the human DPM1 (R92G) *in vivo* mutations^24^ and suggest that the conserved R residue is crucial for GDP-mannose binding. However, these findings could not be fully rationalised by protein homology modeling to existing GT-2 crystal structures, which provide single snapshots of homologous protein structures obtained under specific conditions, nor by other 3D structural predictions, which show a very similar fold for wild-type and mutant enzymes. Crystallisation trials of CU PPMS were unsuccessful, and early NMR studies aimed at probing the protein structure in solution proved challenging, with protein precipitation occurring under the conditions required to acquire amide proton signals (pH 7.0). When the wild-type protein was subjected to circular dichroism (CD) analyses, these revealed an ordered structure with levels of secondary structure close to computed values (Fig. S5); however, no structural changes arising from R to G/A mutations and GDP-mannose binding were detected under the conditions tested. We therefore resorted to the use of molecular dynamics (MD) to simulate protein behaviour.

**Fig. 3:**
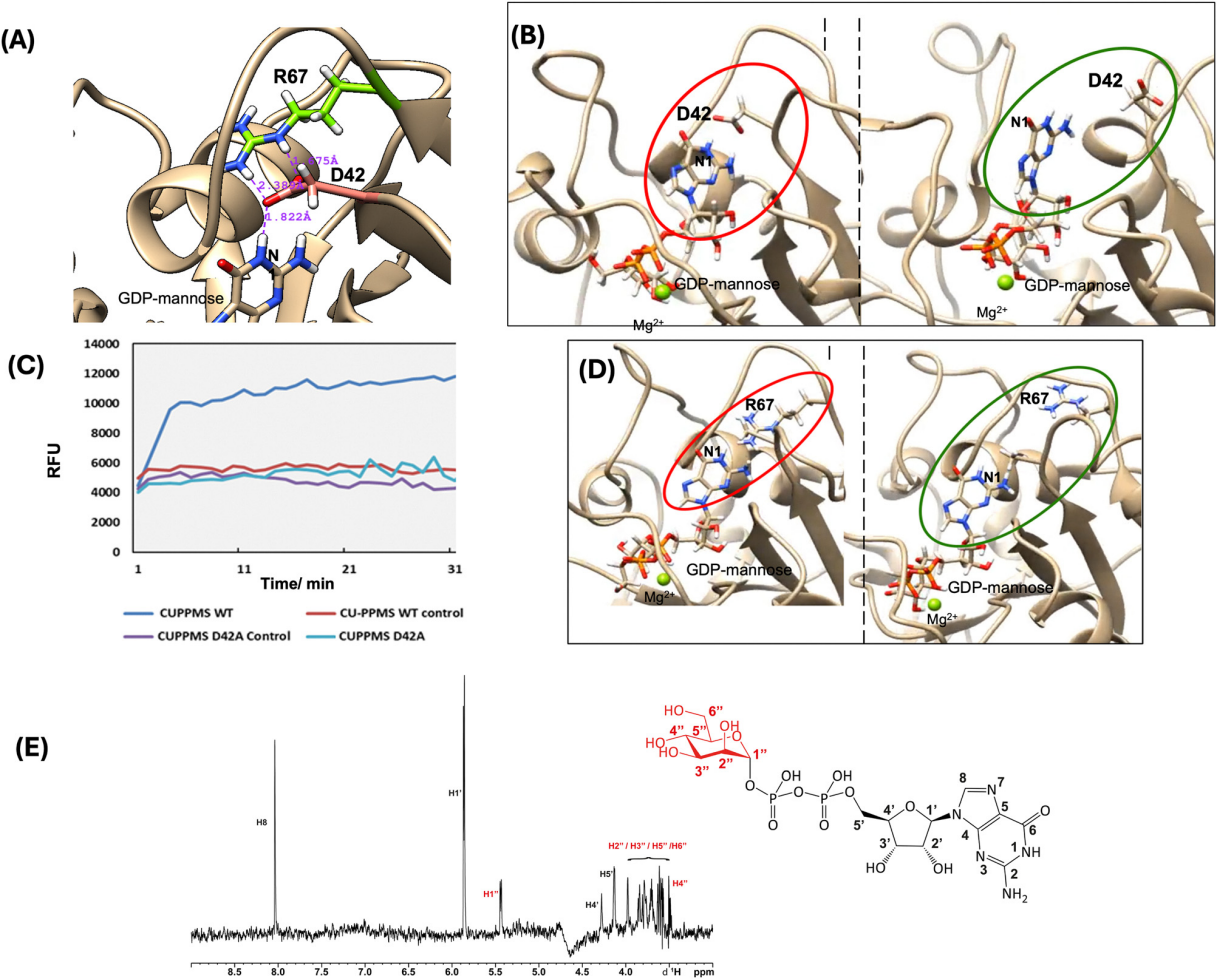
(A) Zoomed in Alphafold model^25^ of the GDP-mannose binding site of CU PPMS (untagged): R67 (green) coordinates D42 (pink), which hydrogen bonds to the N1 of guanosine. (B) Frames from molecular dynamics (MD) simulations of R67G mutant with GDP-mannose bound. Left (red oval): in the initial state D42 is oriented correctly within hydrogen bonding distance to the N1 of guanosine. Right (green oval): over time D42 can rotate away from the active site and GDP-mannose moves away from a catalytic position. (C) Continuous GT activity measured for CU PPMS wild-type and mutants D42A and D98A (details are given in the SI). (D) Selected frames from MD simulations of the D42A mutant with GDP-mannose bound. Left (red oval): the initial state shows R67 located in the correct position; right (green oval): R67 has rotated away from the active site and freely moves around over the course of the simulation; in the absence of hydrogen bonding, the tertiary structure around the GDP-mannose binding site misfolds. (E) ^1^H Saturation Transfer Difference (STD) NMR spectrum of WT CU PPMS acquired in 10 mM sodium phosphate buffer pH 7.5, 50 mM NaCl, 2 mM MgCl_2_, 10% D_2_O and glycerol with GDP-mannose (**1**, structure shown with labelled positions).

The protein tertiary structure was monitored over 1 microsecond of classical MD trajectory. The simulations did not reveal drastic changes in secondary structure, in agreement with the CD data. However, at a molecular level, MD revealed a significant difference in the behaviour of an aspartate (D42) residue, which coordinates R67 and the guanine residue of GDP-mannose within the *C. ulcerans* PPMS (Fig. 3A). In mutant R67G (and similarly in R67A), the lack of the guanidinium side chain causes D42 to lose its position and ‘flip’ around, leading to loss of coordination to GDP-mannose (Fig. 3B). The calculated lifetime of hydrogen bonding between D42 and the N1 of GDP within CU PPMS R67G is indeed drastically reduced (from 65% to 3%, Table S1). To investigate these computational findings at the experimental level, we generated a D42A *C. ulcerans* PPMS mutant: this proved almost inactive (Fig. 3C) and misfolded (Fig. S4), supporting its importance for binding GDP-mannose with the assistance of R67. Parallel MD studies carried out for wild-type human hDPM1 and its reported R92G mutation associated with CDG1e (Table S2) revealed a similar protein behaviour and suggest a similar ‘docking’ role for the conserved R92 in the functioning of DPMSs (SI).

To gather further experimental insights into GDP-mannose binding for CU PPMS, we subjected the WT and R67G mutant proteins (featuring a similar size-exclusion chromatography profile, Fig. 1E and Fig. S4) to ^1^H- and STD-NMR studies in the absence and in the presence of GDP-mannose. Under the utilised conditions (10 mM sodium phosphate buffer, pH 7.5, 50 mM NaCl, 2 mM MgCl_2_, 10% D_2_O and glycerol, 50 µM protein and 5 mM GDP-mannose), we were able to obtain stable protein samples and acquire ^1^H-NMR spectra. Although we could not detect amide proton signals for the proteins nor the N1 proton of guanosine due to their exchange with deuterium, we were able to detect most of the backbone protons of GDP-mannose and some of their changes upon complexation (Fig. 3E and Fig. S6-7). From the collective ^1^H-NMR data acquired, we could observe a stronger interaction between WT CU PPMS and GDP-mannose compared to the R67G mutant case. This is clearly reported by GDP-mannose signal changes upon complexation (*e.g.* mannose H1″ and H2″ coupling constants, Table S3, SI), as well as by STD difference spectra for the protein–ligand complexes (Fig. 3E and S7). These data can be rationalised and integrated also on the basis of our MD simulations, which show a more confined positioning of the mannose residue within the WT protein donor binding site, and, conversely, a wider range of conformations and orientations for mannose in the absence of R67 coordinating D42 (SI). Very recently, new crystal structure data of the archaeal *Pf*DPMS in complex with GDP-mannose and dolichol phosphate mannose have also become available:^26^ in the new structural snapshot, the N1 proton of GDP-mannose coordinates to one oxygen of the side chain of D39 (corresponding to our CU PPMS D42) which also hydrogen bonds to R63 (corresponding to the CU PPMS R67) in a proposed mannosyl ‘pre-transfer’ state. This new evidence validates our MD findings and confirms the importance of CU PPMS R67 (and the conserved arginine residues of homologous proteins) for the ‘docking’ of D42 and the guanosine residue as a prerequisite for efficient transfer of mannose to the polyprenyl phosphate carrier.

## Conclusions

Currently, the workings of many GTs involved in biomolecule functionalisation remain underexplored due to challenging requirements associated with enzyme activity characterisation. The real time monitoring of PPMS enzymes developed herein is in principle applicable to all Leloir GTs, as PNPase enzymes can employ all nucleotide diphosphates to generate RNA for fluorescent detection. Moreover, the new GT activity monitoring approach presented herein has allowed us to shed light on cryptic details of PPMS catalysis leading to glycocarrier formation; in complement with other approaches such as MD simulations and NMR, it holds promise to greatly contribute to a better understanding of carbohydrate processing, with overarching implications for health and disease.

## Materials and methods

### GT activity assays

PNPase/Ribogreen assays were set up in Greiner Bio-One black 96-well plates. Quant-it™ RiboGreen (Life Technologies/Invitrogen) was stored as the manufacturer's stock at 4 °C. 100 µL aliquots of PPMS protein solution (stored at 0.4 mg mL^−1^) were thawed and used immediately as needed. PNPase was stored at −80 °C in 100 µL aliquots at 35.8 µM until needed. Single aliquots were thawed only once. All recombinant enzymes were stored in buffers containing 10% glycerol. Solution A was prepared by diluting 10× PNPase–Ribogreen assay buffer (500 mM Tris pH 7.5, 500 mM NaCl, 0.05% Triton X-100, 1 mM DTT, and 10 mM MgCl_2_) in deionised (18.2 MΩ) water to a 1× concentration; and adding 750 nM PNPase (N435D) enzyme, followed by the stated concentrations of GDP-Mannose (sodium salt from *S. cerevisiae*, Merck), lipid phosphate acceptor (in the range of 50–100 µM, dissolved either in solubilisation buffer containing glycerol or Triton, or, where possible, in HPLC-grade methanol, SI), and 1 : 1600 dilution of Ribogreen. Solution B was prepared by adding 173 nM glycosyltransferase to a 1:10 dilution of 10× PNPase-Ribogreen assay buffer and 18.2 MΩ water. Assay protocol: 25 µL of Solution A were added to 25 µL of Solution B with a multichannel pipette to reach a final assay volume of 50 µL. Plates were immediately added to a Hidex Sense 96 well plate reader. For discontinuous assays, plates were incubated at room temperature (18 °C) for 1 hour and quenched with 10 µL of 8 mM EDTA, pH 8.0, before reading. For continuous assays, the plate was read from time point zero for at least 60 minutes, with each well-read once per minute at 10 flashes per read and an aperture of 1 mm on low lamp power. Wells were excited and read from the top. The excitation and emission wavelengths were 488 nm and 520 nm, respectively. All data analysis was performed in Microsoft Excel. For kinetics analysis, one substrate was kept at a constant concentration (100 µM), whilst the other was serially diluted in seven wells with a ‘blank’ control consisting of buffer alone. All reactions were run at least in triplicates, and data collected as before. Michaelis–Menten kinetic parameters for *K*_m_, *K*_cat_ and *V*_max_ were calculated by using the non-linear least squares regression method using Microsoft Excel with the ‘Solver’ plug-in. The kinetics data from the steady state of the PNPase/Ribogreen assay were converted from µM GDP to picomoles of undecaprenyl phosphomannose (3b) produced per minute, with the aid of the standard curve (Fig. S2C). Observed data were plotted on a separate worksheet, and corresponding expected values were calculated and plotted. The sum of squared residuals (SSR) between observed and expected data was used as the objective function. The Excel Solver add-in was employed to optimise *K*_m_ and *V*_max_ by minimising the SSR. To reduce the risk of convergence on a local minimum, the optimisation was repeated at least ten times using different initial parameter estimates, with manual adjustment between runs to identify the best-fitting solution. Direct PPMS assay product characterisation was carried out from scaled-up enzyme-catalysed reactions of GDP-mannose and lipid phosphate as described in the SI.

### Molecular dynamics

Initial structures (CU PPMS and hDPM1) were generated using homology modelling and Alphafold2,^25^ and each of the mutants was generated with the Rotamers tool in UCSF Chimera.^27^ Initial coordinates for simulation of CU PPMS (WT, R67G, R67A and D42A) and hDPM1 (WT, R92G and D65A) were prepared using AMBER20.^28^ GDP-mannose and Mg^2+^ were modelled in the active site using homologous crystal structures (PDB code 5EKP, 5MM1). Molecular dynamics (MD) simulations were performed using the AMBER ff14SB force field.^29^ GDP-mannose and Mg^2+^ parameters were taken from already available parameter calculations.^30,31^ The resulting structures were neutralized with Cl^−^ ions and solvated with TIP3PBOX, such that no protein atoms were positioned <10–15 Å from any box edge, using the TLEAP program available in the source code of AMBER20.^28,32^ MD heating, equilibration, and production steps were performed using the AMBER 20 software. Simulation used the SHAKE algorithm^33^ to constrain all protein bonds involving a hydrogen atom; a 2.0 fs time-step was used in these simulations.^34^ Long-range electrostatics were calculated using the Particle Mesh Ewald (PME) method with a 12.0 Å cutoff. PME was used for nonbonded interactions. In all simulations, the Langevin thermostat (*γ* = 2.0 ps^−1^) was used to maintain temperature control.^35^ The solvated protein was equilibrated by carrying out a short minimization, 50 ps of heating and 50 ps of density equilibration with weak restraints on the protein, followed by 500 ps of constant pressure equilibration at 300 K. More specifically, after a two-step minimization process, in which solvent molecules were allowed to relax before the entire system was minimized, the system was slowly heated to 300 K over 0.1 ns in a canonical ensemble (NVT) simulation, then equilibrated for 2 ns by performing isothermal–isobaric (NPT) simulations at 300 K using a Berendsen barostat. The production stage runs for a 1 µs using the classical sampling approach.

### ^1^H- and STD-NMR studies

^1^H NMR spectra were recorded on a Bruker Avance 600 MHz spectrometer equipped with a PA BBO 600S3 BB-H-D-05 Z probe utilising the standard Bruker *zgesgp* pulse programme with excitation sculpting for water suppression. Spectra were acquired using 160 scans (*n*_s_ = 160) and 2 dummy scans (*d*_s_ = 2), with a relaxation delay of 2.0 s (*d*_1_ = 2.0 s) and carrier frequency O1P = 4.70 ppm. Experiments were performed at 298 K. NMR samples (250 µL final volume) were prepared from frozen protein samples (WT and R67G CU PPMS) stored as described in the SI and thawed on ice. The samples were buffer exchanged three times with 10 mM sodium phosphate buffer pH 7.5, 50 mM NaCl and 2 mM MgCl_2_ (leaving residual glycerol present for protein stability) ahead of adding D_2_O (10%). Protein final concentration was 50 µM, and GDP-mannose (**1**) was added to protein samples in 100-fold molar excess (5 mM) ahead of data acquisition. ^1^H-NMR spectra of **1** and of WT and R67D mutant proteins were first acquired, followed by spectra of protein:GDP-mannose complexes. Chemical shifts of GDP-mannose were assigned using literature data.^36–38^ Data were processed in TopSpin using standard Fourier transformation and phase and baseline correction.

STD NMR spectra were recorded on a Bruker Avance 600 MHz spectrometer equipped with a PA BBO 600S3 BB-H-D-05 Z probe using the standard Bruker *stddiffesgp* pulse programme with excitation sculpting for water suppression. Experiments were acquired as pseudo-2D datasets with interleaved on- and off-resonance irradiation using 8 scans per increment (*n*_s_ = 8). Selective saturation was applied at 0 ppm (on-resonance) and −40 ppm (off-resonance). Acquisition parameters were as follows: pulprog = *stddiffesgp*, NBL = 2, O1P = 4.70 ppm, and saturation time *d*_20_ = 2.0 s. Spectra were recorded at 298 K in sodium phosphate buffer (pH 7.5) containing 50 mM NaCl, 2 mM MgCl_2_, glycerol and 10% D_2_O as described above. Data were processed in TopSpin by subtraction of the on-resonance spectra from the corresponding off-resonance spectra to afford the STD difference spectra.

## Author contributions

Conceptualisation and writing: RDP and MT; methodology, investigation and data curation: RDP, MT, AG, JL, AC and SM; supervision: MT, AC, JL and AG; resources: MT, JL and AC; funding acquisition for this work: MT.

## Conflicts of interest

There are no conflicts to declare.

## Supplementary Material

CB-007-D6CB00069J-s001

CB-007-D6CB00069J-s002

CB-007-D6CB00069J-s003

CB-007-D6CB00069J-s004

CB-007-D6CB00069J-s005

CB-007-D6CB00069J-s006

CB-007-D6CB00069J-s007

CB-007-D6CB00069J-s008

## Data Availability

The data supporting this article have been included in the supplementary information (SI). Supplementary information: additional materials and methods, supplementary figures and data. See DOI: https://doi.org/10.1039/d6cb00069j.
