# Supplementary material for: Real-time monitoring of glycocarrier formation unravels cryptic details in glycosyl transfer
Source: RSC Chem Biol. 2026 Jun 1;7(7):1382–8. doi: 10.1039/d6cb00069j (PMC13224292; doi:10.1039/d6cb00069j)
Supplement: CB-007-D6CB00069J-s006 [file CB-007-D6CB00069J-s006.pdf]

Supplementary Information (SI) for RSC Chemical Biology.
This journal is © The Royal Society of Chemistry 2026

Real-time monitoring of glycocarrier formation unravels cryptic details in glycosyl transfer

Ryan D. Packer, Angelo Gallo, Alexander D. Cameron, Somnath Mondal, Józef R. Lewandowski and Manuela Tosin*

Supporting Information

Table of contents

Supplementary Figures 3

Fig. S1 (A) Schematic representations of PPMS and DPMS enzyme structures, (B) sequence alignments and (C) homologies…………………………………………….. 3

Fig. S2 PNPAse characterisation……………………………………………4

Fig. S3 WT CU PPMS characterisation………………………………………… 5

Fig. S4 CU PPMS mutant characterisation characterisation…………………………… 6

Fig. S5 CU PPMS structural characterisation ……………………………….…7

Table S1 Molecular dynamics (MDs) simulations for CU PPMS…………………………8

Table S2 Molecular dynamics (MDs) simulations for hDPM1…………………………. 8

Fig. S6 ^{1}H-NMR spectra of GDP-mannose on its own and in complex with WT and R67G CU PPMS… 9

Table S3 Summary of ^{1}H-NMR data for GDP-mannose on its own and in complex……………………. 9

Fig S7 Overlay of STD NMRs………………………………………… …. 10

General materials and methods 11

Table S4 List of plasmids……………………………………………11

Table S5 List of primers…………………………………………… 11

Table S6 List of strains……………………………………………...12

Table S7 List of buffers…..………………………………………….…12

Table S8 PCR conditions………………………………………….…12

Cloning…………………………………………………… 13

Site-directed mutagenesis……………………………………………13

Recombinant protein expression and IMAC purification……………….……………13

Protein Size-Exclusion Chromatography (SEC)……………………………………14

Hydrophobic Interaction Chromatography (HIC)…………………………...…14

Protein characterisation by mass spectrometry……………………………..….14

Circular dichroism analysis……………………………………………...14

In vitro assays of GT activity……………………………………………15

Protein structure modelling……………………………………………...15

Molecular dynamics……………………………………………...15

NMR studies……………………………………………15

Key nucleotide and amino acid sequences……………………………………15

Key plasmid maps……………………………………………..17

References………………………………………… …18

Supplementary Figures

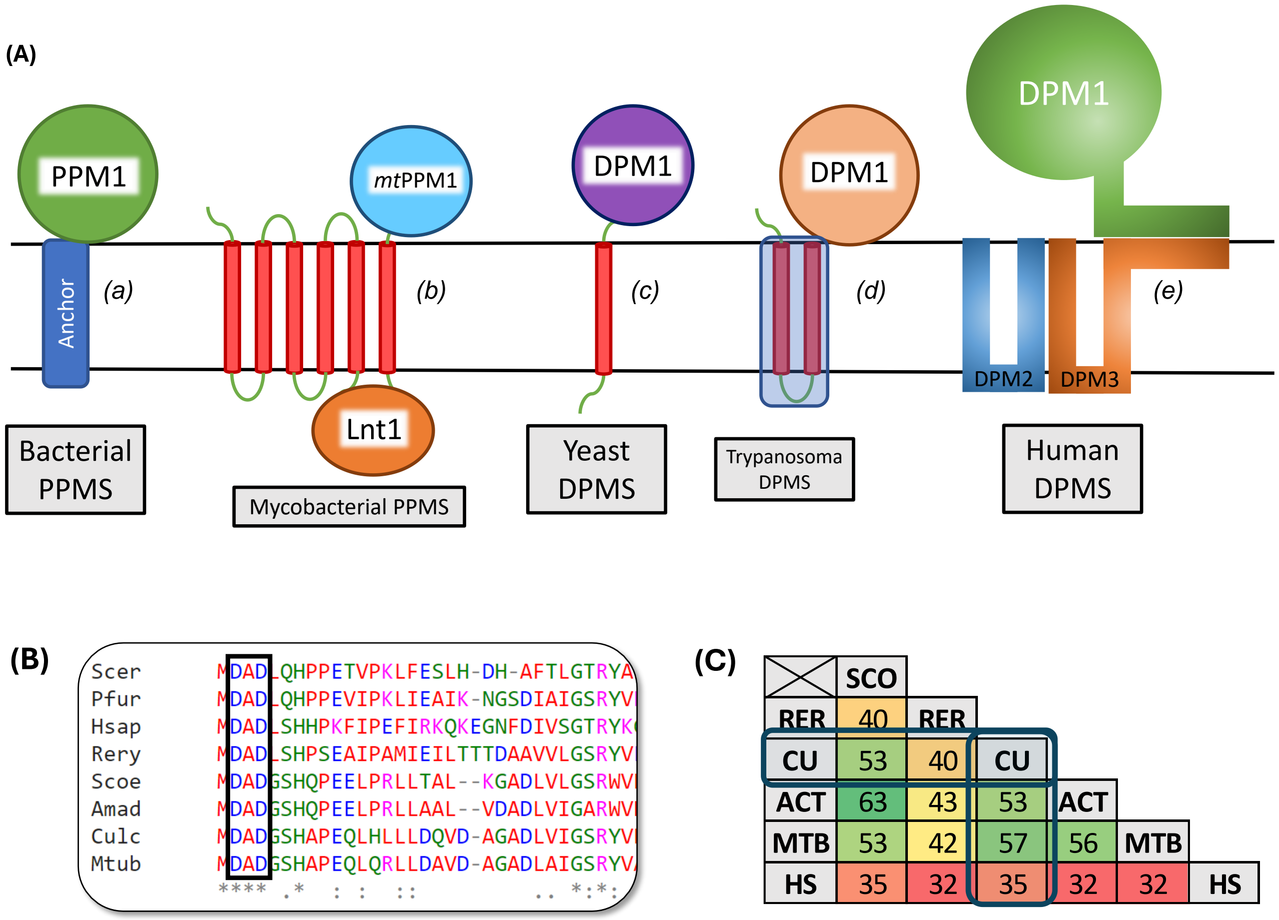

Figure S1 (A) Schematic representations of PPMS and DPMS enzyme structures across different organisms. From left to right: *(a)* a bacterial PPMS, bearing its catalytic site (PPM1) outside the membrane and featuring a transmembrane (or membrane-associated) anchor [1] - archaeal DPMSs present a similar topology; [2] *(b)* a mycobacterial PPMS, featuring a catalytic site (mtPPM1), multi-spanning transmembrane portion and Lnt (lipoprotein N-acyl transferase) activity; [3] *(c)* yeast DPMS, comprising a transmembrane segment and a DPM catalytic site; [4] *(d)* trypanosomal DPMS, featuring a transmembrane portion and DPM catalytic site; [5] *(e)* the human DPMS complex, comprising membrane-integrated accessory proteins DPM2 and DPM3 (accessory proteins) and the catalytic DPM1 protein. [6]
(B) Partial sequence alignment of selected PPMSs (Rery, Scoe, Amad, Culc and Mtb) and DPMSs (Scer, Pfur, Hsap) highlighting the conserved DxD motif within the GT-2 enzymes. **(C)** Sequence homology (in %) of selected PPMS (RER, CU, ACT, MTB) and DPMS (HS) enzymes.

Protein abbreviation legend (with NCBI gene accession number, unless otherwise stated):

Rery or RER = *R. erythropolis* PR4 PPMS (RER_RS20560)
Scoe or SCO = *S. coelicolor* A3(2) PPM1 (SCO1423 or SC6D7.16) [1]
Amad or ACT = *Actinomadura sp.* PPMS (CNF65104)
Culc or CU = *C. ulcerans* PPMS (this study, SQG58706)
Mtub or MTB = *M. tuberculosis H37Rv* PPM1 (Rv2051c) [3]
Scer = *S. cerevisiae* DPM1 (NP_015509) [4]
Pfur = *P. furiosus* DPM1 (UniProt Q8U4M3) [2]
Hsap or HS = *H. sapiens* DPM1 (D86198) [6]

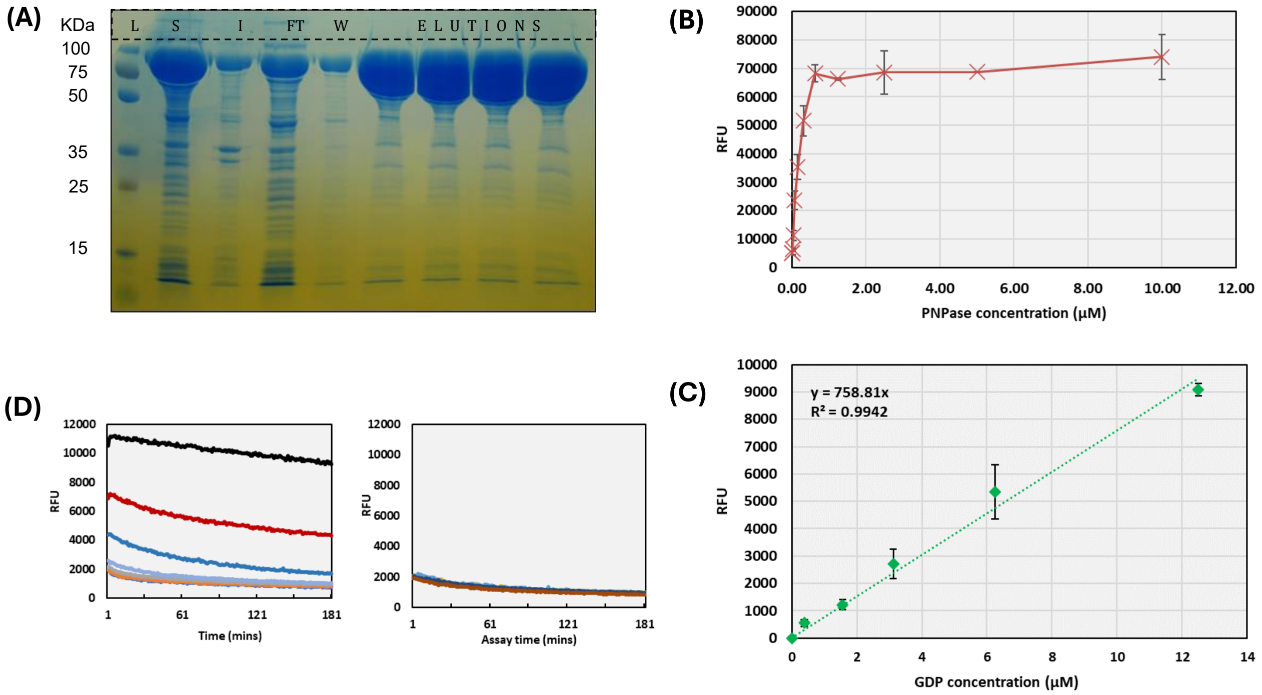

Figure S2 PNPAse characterisation.

(A) 10% SDS-PAGE analysis of recombinant protein overexpression and IMAC purification. From left to right: L= ladder (Protein Plus Prestained, Thermo Fisher), S= soluble, I= insoluble, FT= flow through, W= wash, and eluted fractions.

(B) Effect of varying PNPase enzyme concentration on fluorescent output. GDP was kept at 100 µM in 50 mM Tris-HCl pH 7.5, 50 mM NaCl, 0.005% Triton X-100, 0.1 mM DTT, and 1 mM $MgCl_2$.

(C) Standard curve for PNPase activity and fluorescence upon Ribogreen addition (linear correlation between variable GDP concentration and fluorescence output, keeping PNPAse and Ribogreen concentrations constant in the general assay conditions, see methods; error bars represent standard deviation of 3 replicates).

To confirm the PNPase enzyme activity and, by extension, that the product (PolyG RNA) was responsible for the fluorescent output, an inactive mutant of PNPase (C444W) was generated, as well as an N435D mutant, for which enhanced polymerisation activity over the 3' to 5' exonuclease activity has been reported. [7]

(D) *Left*: PNPase N435D mutant activity plot, showing the fast processing of the mutant at 25 (black), 12.5 (red), 6.25 (blue), 3.13 (cyan), 1.56 (grey), 0.78 (orange) and 0 (green) µM GDP. *Right:* PNPase C444W mutant assayed under identical conditions, displaying no significant fluorescent signal.

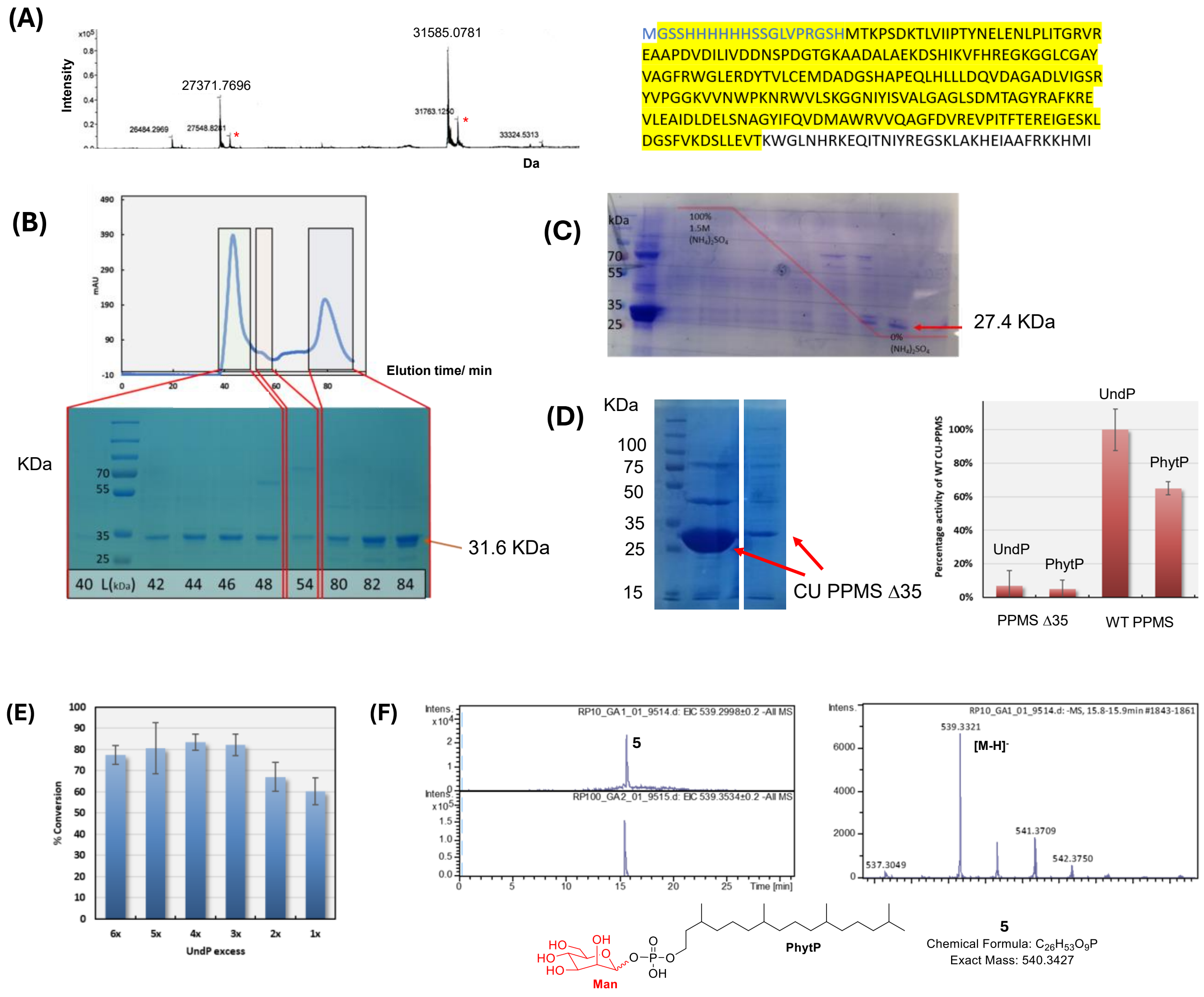

Figure S3 WT CU PPMS characterisation.

(A) ESI-MS analysis (deconvoluted spectrum) of WT CU PPMS mixture, and the expected amino acid sequence (267 amino acids, 31.6 KDa with loss of methionine); the section highlighted in yellow represents the sequence associated to the lower MW band (27.4 KDa, corresponding to a *C*- terminus truncation of 35 amino acids). The hexahistidine tag and thrombin cleavage site are represented in blue in the sequence. Gluconoylated peaks for both protein masses found are indicated in the spectrum with a red asterisk. **(B)** Size exclusion chromatogram (Superdex 200 16/600 column) of IMAC purified WT CU PPMS (top) and accompanying 10% SDS-PAGE analysis (bottom): the upper band purified alone elutes in the aggregate, whereas both bands co-elute as a dimer. **(C)** 10% SDS-PAGE analysis of WT CU PPMS purified *via* HIC (phenyl sepharose column) with a decreasing amount of ammonium sulphate (red line): the lower protein band was isolated in the last fraction (0% ammonium sulphate), however it proved catalytically inactive. To further investigate this: **(D)** a CU PPMS Δ35 C-terminal mutant was generated by site- directed mutagenesis (as later described in general methods): this expressed in *E. coli* as a single protein of approx. 27 KDa (left, concentrated and diluted samples); when tested with UndP and PhytP acceptors it was catalytically inactive (right; enzyme activity normalised against UndP). **(E)** CU PPMS activity tested with excess of undecaprenyl phosphate. GDP-Man was set at a 25 µM concentration and molar excess of undecaprenyl phosphate (UndP, **2b**) was added; upon 1 h incubation, the reaction reached around 85% of GDP-mannose (**1**) conversion to mannosylated UndP (**3b**) (see Methods). **(F)** ESI- MS (negative mode) for mannosylated phytanyl phosphate (**5**), obtained by incubation of synthetic phytanyl phosphate (PhytP) [8] with GDP-mannose in standard reaction conditions without detergent; the product (**5**) was extracted in chloroform/methanol and characterised by LC-HR-ESI-MS (Bruker MaXis **II**, see methods).

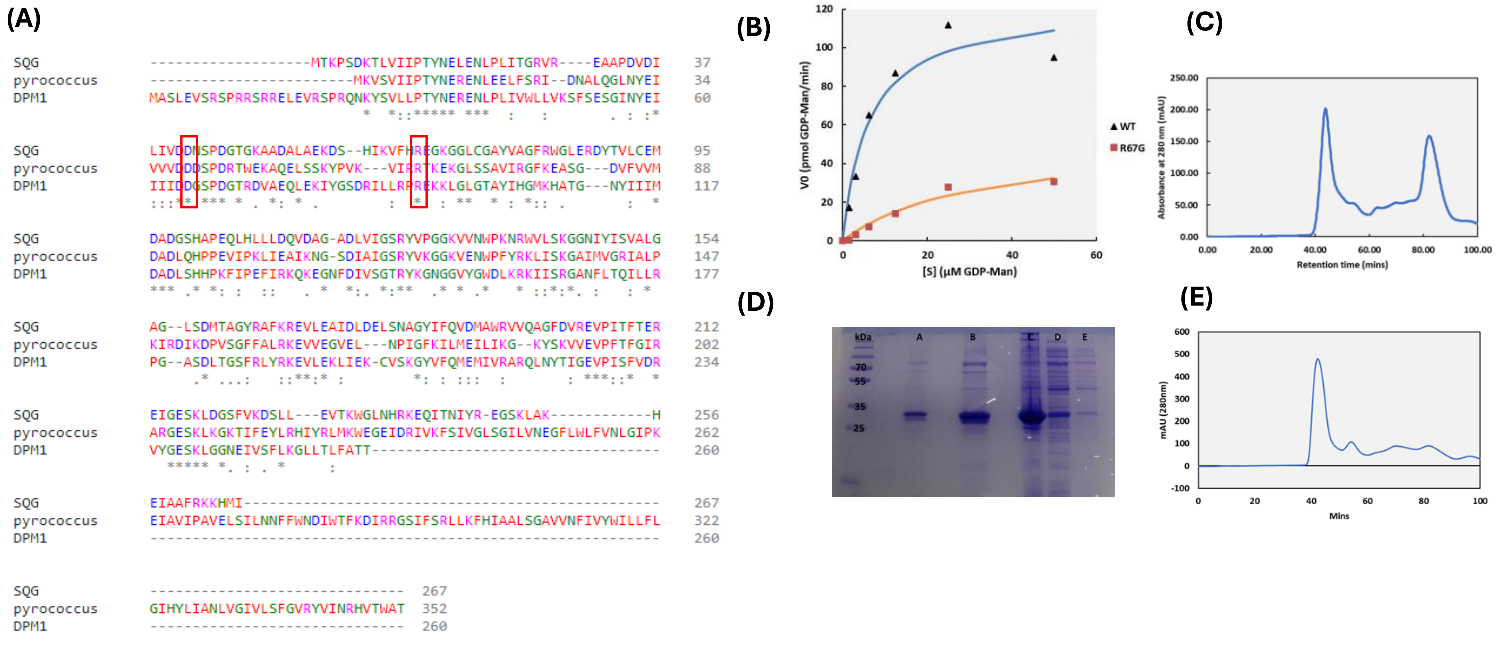

Figure S4 CU PPMS mutant characterisation

(A) Identification of conserved D42 and R67 residues in CU PPMS sequence (SQG, aligned with human DPM1 and *P. furiosus* DPM using Clustal-Omega).

(B) Kinetic analysis of WT and R67G CU PPMS against GDP-mannose (see details in methods).

(C) Size-exclusion chromatography of CU PPMS R67G mutant, mirroring that of the WT enzyme (Fig. 1 and S3).

(D) Expression and purification of CU PPMS D42A mutant (10% SDS-PAGE gel analysis). Left to right: Ladder (Prestained Protein Plus, Thermofisher), A= size-exclusion purified protein, B= IMAC purified protein, C= insoluble fraction, D= flowthrough, E = 20 mM imidazole wash.

(E) Size-exclusion chromatography of CU PPMS D42A mutant. The protein eluted most at 45 min as a large aggregate. The remaining peaks do not appear to form any dominant oligomeric state.

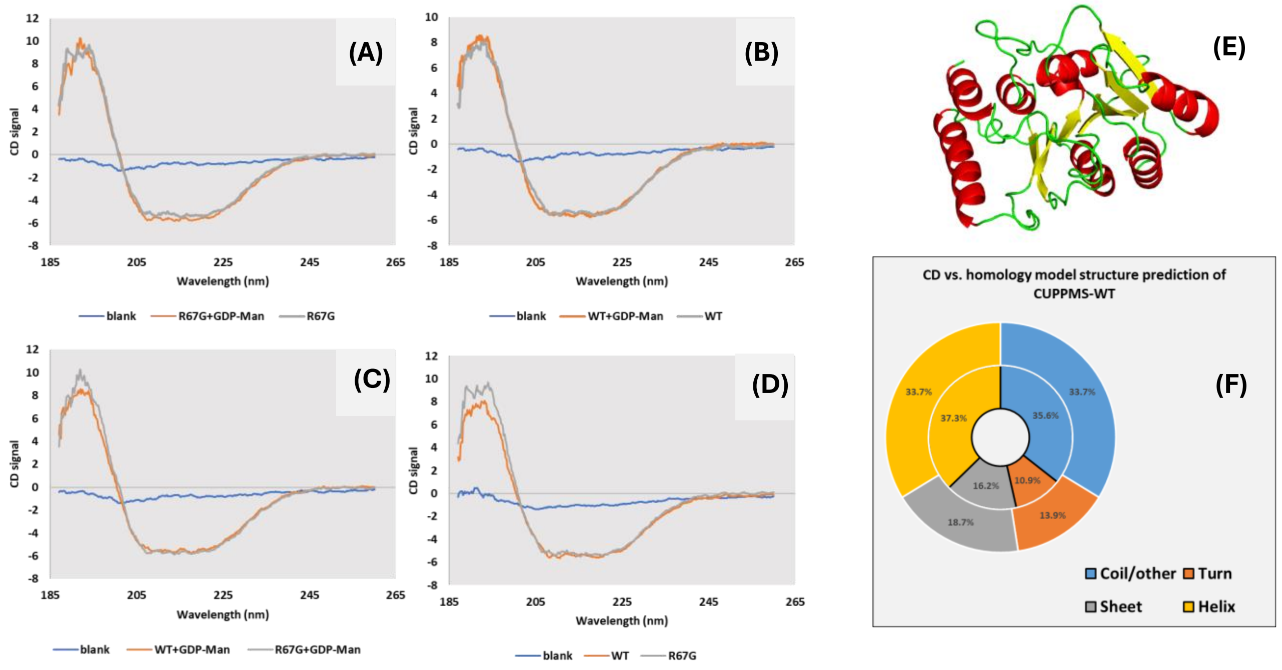

Figure S5 CU PPMS structural characterisation

(A)-(D) CD spectra CD analysis of CU PPMS. **A**: CU PPMS R67G in the presence and in the absence of GDP-mannose. **B**: Wildtype CU PPMS in the presence and in the absence of GDP-mannose. **C**: Wildtype and R67G CU PPMS in the presence of GPD-mannose; and **D**: Wildtype and R67G in the absence of GDP-mannose. All data was acquired over 18 acquisitions in 50 mM sodium phosphate, 50 mM NaCl, 1 mM $MgCl_2$. Regions below 200 nm show variability due to the presence of NaCl. Very few changes are observed between wildtype and R67G or the addition of GDP-mannose. Further experimental details provided in the methods section.

(E) CU PPMS model generated by homology modelling and Alphafold2 [9] (see methods).

(F) Comparison of the *in silico* predicted structural composition of CU PPMS (inner circle) versus CD data acquired over 18 acquisitions (see methods for further details).

Table S1 Molecular dynamics (MD) simulations for CU PPMS

Interaction		H bond lifetime
D42 (wildtype)	R67	67%
D42 (wildtype)	GDP-Man (H1)	65%
D42 – R67G	GDP-Man (H1)	3%

Table S2 Molecular dynamics simulations for hDPM1

Interaction		H bond lifetime
D65 (wildtype)	R92	52%
D65 (wildtype)	GDP-Man (H1)	45%
D65 – R92G	GDP-Man (H1)	5%

PDB views, MD movies and complete list of computed MD parameters for CU PPMS and hDPM1 (wild type and mutants) provided in the additional supplementary zip file.

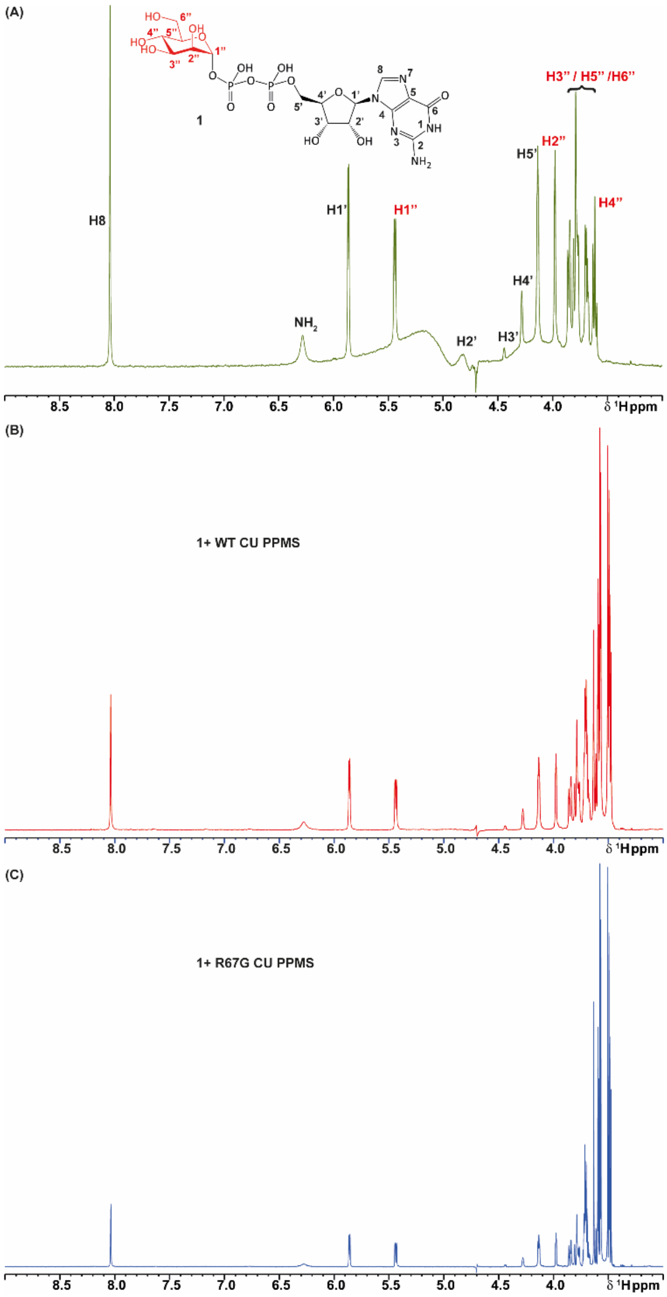

Figure S6 ^{1}H-NMR (600 MHz) of **(A)** GDP-mannose (5 mM) free, **(B)** GDP-mannose (5 mM) in the presence of WT CU PPMS (50 μM) and **(C)** GDP-mannose (5 mM) in the presence of R67G CU PPMS (50 μM). Upon water suppression, H2', H3' and H4' signals are reduced or broadened beyond detection.

Table S3 Summary of ^{1}H-NMR data for GDP-mannose (**1**) on its own and in complex with CU PPMS WT and R67G mutant. Changes occurring upon complex formation are highlighted.

	H8	NH$_2$	H1'	H1''	H5'	H2''	H3''	H5'' + H6$_a$''	H6$_b$''	H4''
1	8.04 (s)	6.28 (b s)	5.86 (d, J 6.1)	5.44 (dd, J 7.8, 1.4)	4.14 (dd, J 5.8, 3.6)	3.98 (b apt t)	3.85 (dd, J 10.0, 3.3)	3.82-3.76 (m)	3.69 (dd, J 12.6, 5.5)	3.61 (t, J 10.1)
1+ **WT**	8.04 (s)	6.28 (b s)	5.86 (d, J 6.1)	5.44 (dd, J 7.8, 1.8)	4.13 (dd, J 5.7, 3.7)	3.98 (b dd, J 3.6, 2.0)	3.85 (dd, J 9.9, 3.4)	3.82-3.76 (m)	overlapping with protein and glycerol signals	3.61 (t, J 10.1) (partially overlapping with protein and glycerol
1+ **R67G**	8.04 (s)	6.28 (b s)	5.86 (d, J 6.1)	5.44 (dd, J 7.7, 1.5)	4.13 (dd, J 5.9, 3.6)	3.98 (b apt t)	3.85 (dd, J 9.9, 3.3)	3.82-3.76 (m)	overlapping with protein and glycerol signals	3.61 (overlapping with protein and glycerol signals)

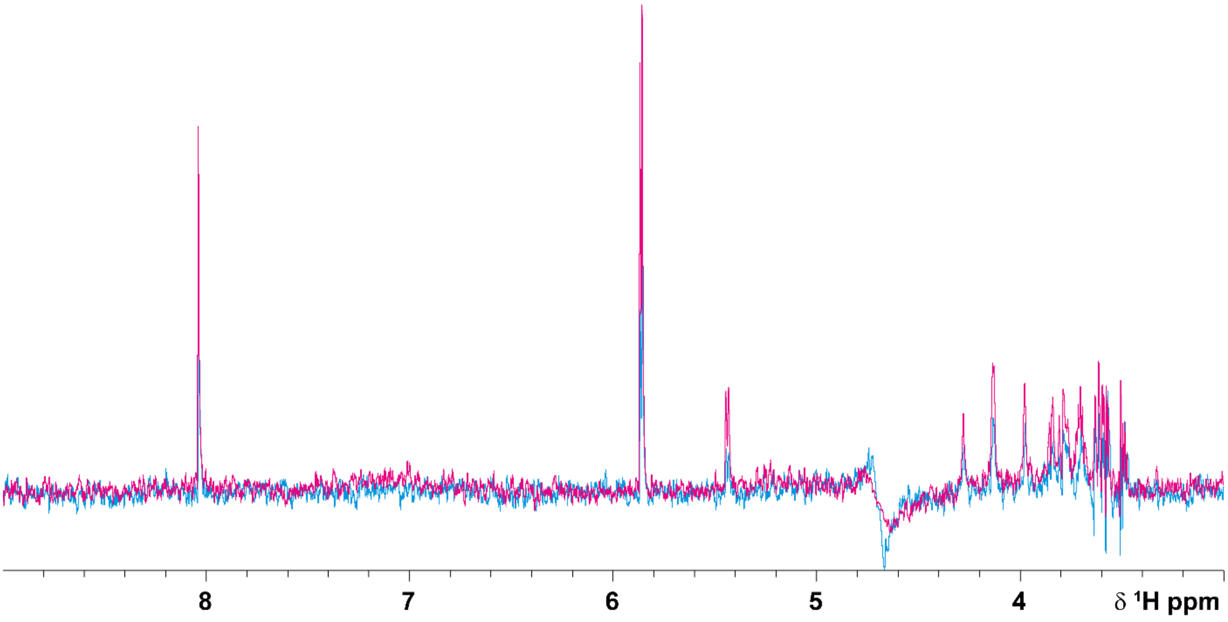

Figure S7 Overlay of ^{1}H STD NMR spectrum of WT CU PPMS (red lines) and ^{1}H STD NMR spectrum of R67G CU PPMS (light blue lines) in complex with **1**.

General materials and methods

All chemicals including antibiotics were purchased from either Merck (UK) or Thermo Fisher Scientific (UK), unless otherwise indicated. Undecaprenyl-MPDA monophosphate was purchased from Larodan. Primers (oligonucleotides) were synthesised by Merck (UK). All biochemicals for molecular biology were purchased from New England Biolabs (NEB UK), Thermo Fisher Scientific (UK) and Invitrogen as indicated. Restriction enzymes (all from NEB) were stored at 20 °C and used with the buffers provided and at suggested temperatures.

Table S4 List of plasmids utilised and generated in this work

Constructs	Origin	Comments
**pET28a(+)**	Novagen	Kan^R, PT7, pBR322 *ori*, N-terminal His$_6$-tag, thrombin cleavage site, for protein expression
**pET28a(+)-pnp WT, C444W and N435D mutants**	WT construct cloned from *E. coli* TOP10 by RDP; mutagenesis performed by RDP	Vectors containing the gene for *E. coli* PNPase used in GT assays with Ribogreen for GDP formation detection and quantification
**pET28a(+)-cuppms WT and mutants**	WT construct designed by RDP and purchased from GenScript; mutagenesis performed by RDP	*Corynebacterium ulcerans* WT PPMS, and R67G/A, D42A and D98A mutants

Table S5 List of primers

Primer name	Sequence (5' to 3')
**pnpFWD-nheI**	AATACATATGCTTAATCCGATCGTTC
**pnpREV-notI**	AACTGCGGCCGCTTACTCGCCCTGTTCA
**PNPC444WF**	GGCTTCCGTGtggGGCGCGTCTC
**PNPC444WR**	ATAGAAGAGGAACCGTTGGATTCAGTGATTTCAGAC
**PNPN435DF**	CACTGAATCCgacGGTTCCTCTT
**PNPN435DR**	ATTTCAGACACAACACGTAC
**SQGR67AF**	AGTGTTCCACgctGAGGGTAAAGGTG
**SQGR67AR**	TTAATGTGGCTATCCTTTTC
**SQGR67GF**	AGTGTTCCACggtGAGGGTAAAG
**SQGR67GR**	TTAATGTGGCTATCCTTTTCC
**SQGD42AF**	GATTGTTGACgcgAACAGCCCGG
**SQGD42AR**	AGGATATCCACGTCCGGC
**SQGD98AF**	AATGGATGCggcTGGTAGCCATG
**SQGD98AR**	TCGCACAGAACGGTATAG
**SQGDelt35F**	GGAAGTGACCtaaTGGGGTCTGA
**SQGDelt35R**	AGCAGGCTATCTTTCACG

Table S6 List of strains

E. coli strain	Comments
**BL21 (DE3)**	Used for protein expression induced by IPTG
**TOP10**	Used for cloning and plasmid amplification

Table S7 List of buffers

All solutions brought to appropriate volumes with 18 milli-Q H_2O unless otherwise specified.

Buffer name	Purpose	Contents
**Buffer A**	Cell lysis	25 mM Tris HCl, 300 mM NaCl, 20 mM Imidazole, 5% glycerol, pH 8.0; stored at RT
**Buffer B**	IMAC elution	25 mM Tris HCl, 300 mM NaCl, 300 mM Imidazole, 5% glycerol, pH 8.0; stored at RT
**Buffer C**	Gel filtration/SEC	50 mM Tris HCl, 150 mM NaCl, 5% glycerol, pH 8.0; stored at – 4°C
**Buffer D**	Cell lysis	50 mM Tris HCl, 300 mM NaCl, 10 mM $CaCl_2$, 20 mM Imidazole, 5% glycerol, pH 8.0; stored at RT
**Buffer E**	IMAC elution	50 mM Tris HCl, 300 mM NaCl, 10 mM $CaCl_2$, 300 mM Imidazole, 5% glycerol, pH 8.0; stored at RT
**Buffer F**	CD	50 mM Na_2HPO_4, 1 mM $MgCl_2$, pH 8.0
**Buffer G**	Protein MS	100 mM Ammonium acetate
**Buffer H/ HIC start buffer**	HIC Chromatography	1.5 M Ammonium sulfate, 50 mM sodium Tris-HCl, pH 8.0. Elution buffer (end Buffer) was 50 mM Tris-HCl, pH 8.0
**10x PNPase-Ribogreen assay buffer**	Ribogreen assays	500 mM Tris pH 7.5, 500 mM NaCl, 1 mM DTT, 10 mM $MgCl_2$, 0.05% Triton X-100
**10X Lipid phosphate solubilisation buffer**	Solubilising lipid phosphates for assays/analysis	500 mM Tris pH 7.5, 20% glycerol or 2% Triton X-100 (sonicated and warmed at 37°C for 15 min) or 100% methanol for phytanyl phosphate
**Buffer I**	Protein NMR	10 mM sodium phosphate, 50 mM NaCl, 2 mM $MgCl_2$, 10% D_2O, glycerol, pH 7.5.

Table S8 PCR conditions used in this work

The reaction cycled between denaturation and extension for 25-35 cycles as required.
Annealing temperatures were calculated using the NEB Tm Calculator website.

Step	Temp (°C)	Time (s)
**Initial denaturation**	98	60
**Denaturation**	98	10
**Annealing**	See primers in Table S2	15
**Extension**	72	40
**Final Extension**	72	120
**Hold**	4	infinite

Cloning

Unless otherwise stated, PCR was performed with Q5 Polymerase (NEB) with ThermoFisher restriction enzymes in a Bio-Rad thermocycler and 0.5 mL thin-walled PCR tubes. DNA sequences with a GC content above 60% were run with GC Enhancer according to the NEB protocol. All PCR was run according to the conditions reported in Table S7.

The *pnp* gene [7] was amplified from *E. coli* TOP10 genomic DNA, using the primers pnp-forward and pnp-reverse (Table S5) and Q5 High-Fidelity DNA Polymerase (NEB) per manufacturer's protocol, under the PCR conditions reported (Table S8). The resulting PCR product (analysed by agarose gel electrophoresis and subsequently purified using Monarch DNA gel extraction kit from NEB) was digested with NcoI and XhoI and inserted into pET28a(+) digested with the same restriction enzymes. Ligation reactions were performed using ANZA ligase 4x master mix (Invitrogen). An aliquot of assembled reactions was used for transformation of competent *E. coli* TOP10. A single colony was inoculated into LB medium supplemented with the appropriate antibiotic (50 μg/mL for kanamycin) for plasmid miniprep (GeneJet kit by ThermoFisher) and sequencing. Inserts were confirmed by Sanger sequencing by Eurofins/GATC using the T7 forward and pET-RP commercial primers available at Eurofins/GATC.

Site-directed mutagenesis

Mutagenesis reactions were carried out using Q5 Site-Directed Mutagenesis Kit (NEB) in accordance with the manufacturer's recommended protocol. The primers were designed using NEBase Changer website (http://nebasechanger.neb.com/). Cycles were repeated 35 times followed by treatment with the KLD mixture in the kit and transformed into *E. coli* TOP10 for plasmid miniprep and sequencing as described above.

Recombinant protein expression and IMAC purification

100 ng of plasmid DNA was added to 50 μL of *E. coli* BL21 (DE3) and left to incubate on ice for 30 mins. Cells were heat-shocked at 42°C for 45 seconds and placed back on ice for 5 minutes. 1 mL of Luria Bertani (LB) or SOC media was added to the cell mixture and incubated at 37°C for 1 hour shaking at 180 rpm. 50 μL of the cell mixture were spread onto LB-agar plates containing kanamycin (50 μg/mL) and left to incubate at 37°C overnight. Single colonies were selected and inoculated into a 5 mL LB starter culture (10 g/ L tryptone/peptone, 10 g/ L NaCl, 5 g/ L yeast extract) supplemented with 50 μg/mL antibiotic and left to grow for at least six hours or overnight. These starter cultures were used to inoculate 1:4 media to air LB expression cultures in 1:100 dilutions supplemented with kanamycin (50 μg/mL). The expression cultures were left to grow at 37°C and 180 rpm to an OD600 of 0.6 and induced with IPTG to a final concentration of 0.5 mM. All constructs were left to express overnight at 15°C or at 30°C for 3 hours. Expression cultures were spun down at 10,000 x g for 20 minutes at 4°C and resuspended in buffer A (for PNPAse expression) or D (for CU PPMS expression, Table S7). The cell suspensions were lysed with a French press at 20 kpsi and spun down at 40,000 x g for 20 minutes at 4°C. The supernatant was collected, filtered through a Sartorius 0.22 μm filter and added to cobalt-talon or nickel-NTA resin equilibrated with buffer A or D and left to incubate on a rotary table for 1 hour at 4°C. Alternatively the lysate mixture was added to a gravity flow column loaded with nickel-Sepharose (Fast Flow, Sigma-Aldrich) and a frit at 4°C and left to flow through the column. 5 resin volumes of buffer A/D were added to the column as a wash, followed by 10 resin volumes of elution buffer B (for PNPAse) or E (for CU PPMS), and the eluent collected. Protein expression was confirmed by 10% SDS-PAGE analyses.

Protein concentration was estimated using a nanodrop Lite UV-VIS reading absorption at 280nm, measuring 1 µL of eluent against 1 µL of elution buffer. Alternatively, protein concentration was estimated by using the Bradford method. [10] All recombinant enzymes were stored in Buffer C with no detergent and 10% glycerol.

Protein Size Exclusion Chromatography (SEC)

The eluent from IMAC was concentrated to 2.5 mL using Sartorius 10 kDa concentrators and loaded onto a Superdex 200 16/600 column equilibrated with buffer C. The protein was eluted through the column at 1 mL/min and collected in 1 mL fractions at room temperature. All SEC was performed on an AKTA Explorer and Unicorn 5.1 software; data were analysed by Microsoft Excel.

Hydrophobic Interaction Chromatography (HIC)

Protein samples from SEC were concentrated to 2.5 mL and buffer exchanged into buffer H using a PD-10 column. A GE Healthcare 5 mL phenyl sepharose column was preequilibrated with Buffer H (Table S7). 5 mL of protein sample was loaded onto the column before washing with two column volumes of Buffer H. The protein was then eluted stepwise in column volume increments of 50 mM Tris pH 8.0 using an AKTA Explorer instrument. Fractions were analysed by 10% SDS-PAGE.

Protein characterisation by mass spectrometry

Purified recombinant proteins were prepared for MS analysis by buffer exchanging in 100 mM ammonium acetate pH 8.0 (buffer G) *via* a PD-10 column and Sartorius 10 kDa cutoff concentrators to a 1 mg/ mL. The samples were analysed on Bruker MaXis II electrospray ionisation time-of-flight mass spectrometer (ESI-TOF-MS) using a Dionex 3000 RS UHPLC fitted with an ACE C4-300 RP column (100 x 2.1 mm, 5 µml; flow rate 0.2 mL/min, eluting with water and acetonitrile containing 0.1% formic acid).

Circular dichroism analysis

SEC purified protein was buffer exchanged into buffer F (Table S7); solid debris was removed by centrifugation prior to the addition of the protein to a U-shaped Circular Dichroism cuvette. 200 microlitres of protein solution at 0.1 mg/mL was added to the cuvette (determined by optimisation of varying protein concentration between 0.01 and 1 mg/mL), and the cuvette was placed into the CD reader slot of a JASCO J-1500 CD Spectrometer. The machine was pre-flushed with N_2 gas and kept under N_2 for the duration of the experiments. Mg^{2+} and GDP-mannose were added as required at concentrations of 0.1 mM. CD, HT (High Tension) and absorbance channels were used to measure data. Data were collected between 260 nm and 180 nm in a continuous scan mode with a speed of 100 nm/min for 18 acquisitions per sample and blank, plus one calibration acquisition to normalise the axes at the beginning of each run. The CD and FL scales were 200 mdeg/1.0 dOD and the digital integration time was 1 second. The cell length was retained at 10 mm; data were analysed using the online server DichroWeb.[11]

In vitro assays of GT activity

PNPase/Ribogreen assays and kinetics analysis were run as described in the article text. Direct PPMS assay product characterisation was carried out from scaled-up enzyme- catalysed reactions of GDP-mannose and phytanyl phosphate [8] (0.5 mL assay volume, no detergent present) extracted with chloroform and methanol mixtures (1:1, 0.5 mL), followed by TLC analysis (in 75 $CHCl_3$: 25 MeOH : 3.6 dH_2O: 0.4 NH_4OH, product **5** staining with permanganate and molybdenum blue) and LC-HR-ESI-MS analysis of the organic extract ($C_{26}H_{53}O_9P$, expected *m/z* 539.3354 for $[M-H]^-$, *m/z* 539.3321 found- see Fig. S3 F).

Protein structure modelling

Nucleotide sequences for constructs of interest were obtained from the NCBI GenBank service. They were converted to amino acid sequences *via* the ExPasy translate tool. [12] Amino acid sequences were then entered into the SwissModel/Phyre2 with default settings and modelled on *pf*DPMS/GtrB. Alphafold structures were acquired through the plug-in with ChimeraX. Models were analysed for their structural features with Chimera and ChimeraX, with *pf*DPMS superimposed with the MatchMaker plug-in to align the 3D structures.

Molecular dynamics

These have been described in the article text. PDB views, MD movies and complete list of computed MD parameters for CU PPMS and hDPM1 (wild type and mutants) provided in the additional supplementary zip file.

NMR studies

These have been described in the article text. Raw files are available at:
https://doi.org/10.5281/zenodo.20141986

Key nucleotide and amino acid sequences:

>CULC22_RS05480(CUPPMS)

```
ATGACCAAGCCGAGCGACAAAACCCTGGTGATCATTCCGACCTACAACGAGCTGGAAAACCTGCCGCTGATCACCGGTCG
TGTTCGTGAAGCGGCGCCGGACGTGGATATCCTGATTGTTGACGATAACAGCCCGGATGGTACCGGCAAGGCGGCGGATG
CGCTGGCGGAAAAGGATAGCCACATTAAAGTGTTCCACCGTGAGGGTAAAGGTGGCCTGTGCGGTGCGTACGTGGCGGGT
TTTCGTTGGGGCCTGGAGCGTGACTATACCGTTCTGTGCGAAATGGATGCGGATGGTAGCCATGCGCCGGAGCAGCTGCA
CCTGCTGCTGGACCAAGTTGATGCGGGTGCGGATCTGGTGATCGGCAGCCGTTACGTTCCGGGTGGCAAGGTGGTTAACT
GGCCGAAAAACCGTTGGGTGCTGAGCAAGGGTGGCAACATCTACATTAGCGTTGCGCTGGGTGCGGGTCTGAGCGACATG
ACCGCGGGCTATCGTGCGTTCAAACGTGAGGTGCTGGAAGCGATCGACCTGGATGAACTGAGCAACGCGGGTTATATTTT
TCAGGTTGACATGGCGTGGCGTGTGGTTCAAGCGGGCTTCGATGTGCGTGAAGTTCCGATCACCTTTACCGAGCGTGAAA
TTGGCGAGAGCAAGCTGGACGGCAGCTTCGTGAAAGATAGCCTGCTGGAAGTGACCAAGTGGGGTCTGAACCACCGTAAA
GAGCAGATCACCAACATTTATCGTGAAGGCAGCAAGCTGGCGAAACACGAGATCGCGGCGTTTCGTAAGAAACACATGAT
TTAA
```

>CULC22_RS05480(CUPPMS)

MTKPSDKTLVIIPTYNELENLPLITGRVREAAPDVDILIVDDNSPDGTGKAADALAEKDSHIKVFHREGKGGLCGAYVAG
FRWGLERDYTVLCEMDADGSHAPEQLHLLLDQVDAGADLVIGSRYVPGGKVVNWPKNRWVLSKGGNIYISVALGAGLSDM
TAGYRAFKREVLEAIDLDELSNAGYIFQVDMAWRVVQAGFDVREVPITFTEREIGESKLDGSFVKDSLLEVTKWGLNHRK
EQITNIYREGSKLAKHEIAAFRKKHMI*

Key amino acids for this study: DAD (96-98; DxD motif); D42; R67

>pnp(PNPase)

ATGCTTAATCCGATCGTTCGTAAATTCCAGTACGGCCAACACACCGTGACTCTGGAAACCGGCATGATGGCTCGTCAGGC
TACTGCCGCTGTTATGGTTAGCATGGATGACACCGCGGTATTCGTTACCGTTGTTGGCCAGAAAAAAGCCAAACCAGGTC
AGGACTTCTTCCCACTGACCGTTAACTATCAGGAGCGTACCTACGCTGCTGGTCGTATCCCGGGTAGCTTCTTCCGTCGT
GAAGGCCGCCCAAGCGAAGGCGAAACCCTGATCGCGCGTCTGATTGACCGCCCGATTCGCCCGCTGTTCCCGGAAGGCTT
CGTCAACGAAGTTCAGGTTATCGCCACCGTGGTTTCTGTTAACCCGCAAGTTAACCCGGATATCGTCGCGATGATTGGTG
CTTCCGCAGCGCTGTCTCTGTCTGGTATTCCGTTCAATGGCCCGATTGGTGCTGCCCGCGTAGGTTACATCAATGACCAG
TACGTACTGAACCCGACTCAGGACGAGCTGAAAGAGAGCAAACTGGATCTGGTTGTTGCCGGTACTGAAGCCGCTGTACT
GATGGTTGAATCTGAAGCTCAACTGCTGAGCGAAGACCAGATGCTGGGCGCAGTAGTGTTCGGTCATGAACAACAGCAGG
TTGTTATTCAGAACATCAATGAACTGGTGAAAGAAGCCGGTAAACCGCGTTGGGACTGGCAGCCGGAGCCGGTAAACGAA
GCGCTAAACGCGCGCGTTGCTGCACTGGCTGAAGCTCGCCTGAGCGATGCTTACCGCATCACCGACAAACAAGAGCGTTA
TGCGCAGGTTGATGTCATCAAATCTGAAACCATCGCGACGCTGCTTGCTGAAGACGAAACCCTGGACGAAAACGAACTGG
GTGAAATTCTGCACGCGATCGAGAAAAACGTTGTTCGTAGCCGCGTACTGGCAGGCGAACCGCGTATCGACGGTCGTGAA
AAAGATATGATCCGTGGTCTGGATGTGCGTACTGGCGTGCTGCCGCGTACTCACGGTTCTGCGCTGTTCACCCGTGGTGA
AACGCAGGCGCTGGTTACCGCAACGCTGGGTACTGCTCGTGACGCGCAGGTTCTTGATGAACTGATGGGCGAACGTACCG
ATACCTTCCTGTTCCACTACAACTTCCCTCCGTACTCCGTAGGCGAAACCGGCATGGTCGGTTCTCCGAAGCGTCGTGAA
ATTGGTCACGGTCGTCTGGCGAAGCGCGGCGTGCTGGCAGTCATGCCGGATATGGACAAATTCCCGTACACCGTACGTGT
TGTGTCTGAAATCACTGAATCCAACGGTTCCTCTTCTATGGCTTCCGTGTGCGGCGCGTCTCTGGCGCTGATGGACGCAG
GTGTGCCGATCAAAGCTGCCGTTGCGGGTATCGCAATGGGTCTGGTGAAAGAAGGCGACAACTACGTTGTACTGTCTGAC
ATTTTGGGCGACGAAGATCACCTGGGCGATATGGACTTCAAAGTTGCAGGTTCCCGCGACGGTATCTCTGCACTGCAGAT
GGATATCAAAATTGAAGGTATCACCAAAGAGATCATGCAGGTTGCGCTGAACCAGGCTAAAGGTGCGCGTCTGCATATCC
TGGGCGTAATGGAACAGGCGATCAACGCGCCGCGTGGCGATATCTCTGAGTTCGCACCGCGTATCCATACCATCAAGATC
AACCCGGACAAGATCAAAGATGTTATCGGTAAAGGCGGCTCTGTTATCCGTGCCCTGACCGAAGAAACCGGCACCACCAT
CGAAATCGAAGATGACGGTACTGTGAAGATCGCAGCGACCGACGGCGAGAAAGCGAAACATGCTATTCGTCGTATCGAAG
AGATCACTGCAGAAATCGAAGTGGGCCGCGTCTACACTGGTAAAGTGACCCGTATCGTTGACTTTGGCGCATTTGTTGCC
ATCGGCGGCGGTAAAGAAGGTCTGGTCCACATCTCTCAAATCGCTGACAAACGCGTTGAGAAAGTGACCGATTACCTGCA
GATGGGTCAGGAAGTACCGGTGAAAGTTCTGGAAGTTGATCGCCAGGGCCGTATCCGTCTGAGCATTAAAGAAGCGACTG
AGCAGTCTCAACCTGCTGCAGCACCGGAAGCTCCGGCTGCTGAACAGGGCGAGTAA

> pnp(PNPase)

MLNPIVRKFQYGQHTVTLETGMMARQATAAVMVSMDDTAVFVTVVGQKKAKPGQDFFPLTVNYQERTYAAGRIPGSFFRR
EGRPSEGETLIARLIDRPIRPLFPEGFVNEVQVIATVVSVNPQVNPDIVAMIGASAALSLSGIPFNGPIGAARVGYINDQ
YVLNPTQDELKESKLDLVVAGTEAAVLMVESEAQLLSEDQMLGAVVFGHEQQQVVIQNINELVKEAGKPRWDWQPEPVNE
ALNARVAALAEARLSDAYRITDKQERYAQVDVIKSETIATLLAEDETLDENELGEILHAIEKNVVRSRVLAGEPRIDGRE

KDMIRGLDVRTGVLPRTHGSALFTRGETQALVTATLGTARDAQVLDELMGERTDTFLFHYNFPPYSVGETGMVGSPKRRE
IGHGRLAKRGVLAVMPDMDKFPYTVRVVSEITESNGSSSMASVCGASLALMDAGVPIKAAVAGIAMGLVKEGDNYVVLSD
ILGDEDHLGDMDFKVAGSRDGISALQMDIKIEGITKEIMQVALNQAKGARLHILGVMEQAINAPRGDISEFAPRIHTIKI
NPDKIKDVIGKGGSVIRALTEETGTTIEIEDDGTVKIAATDGEKAKHAIRRIEEITAEIEVGRVYTGKVTRIVDFGAFVA
IGGGKEGLVHISQIADKRVEKVTDYLQMGQEVPVKVLEVDRQGRIRLSIKEATEQSQPAAAPEAPAAEQGE*

Key amino acids for this study: N435; C444

Key plasmid maps

pET28a-*pnp*

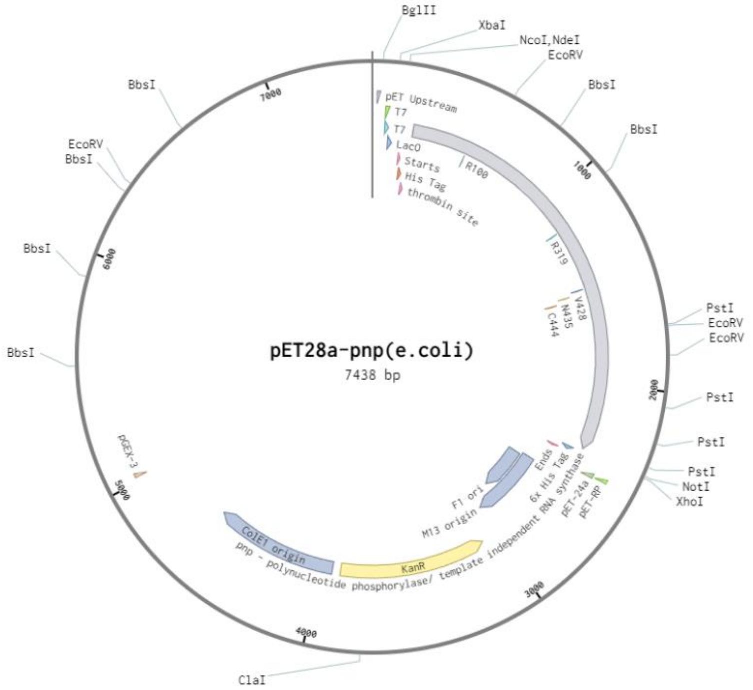

pET28a(+)-*cuppms*-WT and mutants

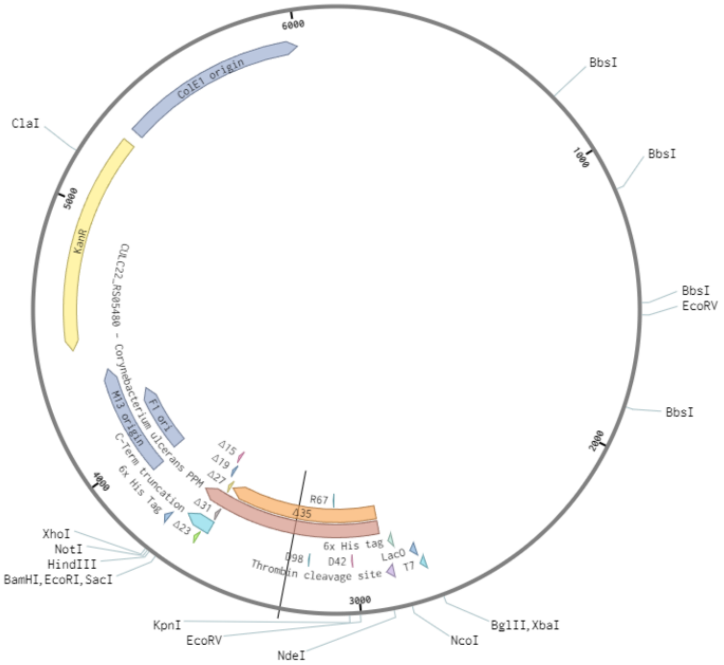

References

[1] D. A. Cowlishaw M. C. Smith, *J. Bacteriol.* **2002**, *184*, 6081–6083.

[2] R. Gandini, T. Reichenbach, T.-C. Tan, C. Divne, *Nat. Commun.* **2017**, *8*, 120.

[3] S. S. Gurcha, A. R. Baulard, L. Kremer, C. Loch, D. B. Moody, W. Muhlecker, C. E. Costello, D. C. Crick, P. J. Brennan, G. S. Besra, *Biochem. J*. **2002**, *365,* 441-450.

[4] P. Orlean, C. Albright, P. W. Robbins, *J. Biol. Chem*. **1988**, 263, 17499–17507.

[5] R. Mazhari-Tabrizi, V. Eckert, M. Blank, R. Müller, D. Mumberg, M. Funk, R. T. Schwarz, *Biochem. J.* **1996**, *316*, 853–858.

[6] Y. Maeda, S. Tanaka, J. Hino, K. Kangawa, T. Kinoshita, *EMBO J*. **2000**, *19*, 2475-2482.

[7] A.-C. Jarrige, D. Bréchemier-Baey, N. Mathy, O. Duché, C. Portier, *J. Mol. Biol*. **2002**, *321*, 397–409.

[8] I. Sprung, L. Carmés, G. M. Watt, S. L. Flitsch, *ChemBioChem* **2003**, *4*, 319- 332.

[9] J. Jumper, R. Evans, A. Pritzel, *et al. Nature* **2021**, *596*, 583–589.

[10] M. M. Bradford, *Anal. Biochem*. **1976**, *72*, 248-254.

[11] A. Lobley, L. Whitmore, B. A. Wallace, *Bioinformatics* **2002**, *18*, 211–212.

[12] E. Gasteiger, A. Gattiker, C.Hoogland, I. Ivanyi I, R. D. Appel, A. Bairoch, *Nucleic Acids Res*. **2003**, *31*, 3784-3788.
